# Effects of a Postbiotic and Prebiotic Mixture on Suckling Rats’ Microbiota and Immunity

**DOI:** 10.3390/nu13092975

**Published:** 2021-08-27

**Authors:** Carla Morales-Ferré, Ignasi Azagra-Boronat, Malén Massot-Cladera, Sebastian Tims, Karen Knipping, Johan Garssen, Jan Knol, Àngels Franch, Margarida Castell, María J. Rodríguez-Lagunas, Francisco J. Pérez-Cano

**Affiliations:** 1 Physiology Section, Department of Biochemistry and Physiology, Faculty of Pharmacy and Food Science, University of Barcelona (UB), 08028 Barcelona, Spain; carla.moralesferre@ub.edu (C.M.-F.); ignasiazagra@ub.edu (I.A.-B.); malen.massot@ub.edu (M.M.-C.); angelsfranch@ub.edu (À.F.); margaridacastell@ub.edu (M.C.); franciscoperez@ub.edu (F.J.P.-C.); 2 Nutrition and Food Safety Research Institute (INSA-UB), 08921 Santa Coloma de Gramenet, Spain; 3 Danone Nutricia Research, 3584 CT Utrecht, The Netherlands; sebastian.tims@danone.com (S.T.); karen.knipping@danone.com (K.K.); johan.garssen@danone.com (J.G.); jan.knol@danone.com (J.K.); 4 Division of Pharmacology, Utrecht Institute for Pharmaceutical Sciences, Faculty of Science, Utrecht University, 3584 CA Utrecht, The Netherlands; 5 Laboratory of Microbiology, Wageningen University, 6708 PB Wageningen, The Netherlands

**Keywords:** postbiotic, prebiotic, suckling rats, Lactofidus, scGOS/lcFOS, microbiota, SCFA

## Abstract

Human milk serves as a model for infant formula providing nutritional solutions for infants not able to receive enough mother’s milk. Infant formulas aim to mimic the composition and functionality of human milk by providing ingredients reflecting those of the latest human milk insights, such as prebiotics, probiotics and postbiotics. The aim of this study was to examine the effects of the supplementation with a postbiotic (Lactofidus^TM^) and its combination with the prebiotics short-chain galactooligosaccharides (scGOS) and long-chain fructooligosaccharides (lcFOS) in a preclinical model of healthy suckling rats. Pups were supplemented daily with Lactofidus^TM^ (POST group) and/or scGOS/lcFOS (P+P and PRE groups, respectively). Body weight and fecal consistency were analyzed. At the end of the study, immunoglobulin (Ig) profile, intestinal gene expression, microbiota composition and short chain fatty acid (SCFA) proportion were quantified. The supplementation with all nutritional interventions modulated the Ig profile, but the prebiotic mixture and the postbiotic induced differential effects: whereas scGOS/lcFOS induced softer feces and modulated microbiota composition and SCFA profile, Lactofidus™ upregulated Toll-like receptors gene expression. The use of the combination of scGOS/lcFOS and Lactofidus™ showed the effects observed for the oligosaccharides separately, as well as showing a synergistic impact on animal growth. Thus, the combined use of both products seems to be a good strategy to modulate immune and microbial features in early life.

## 1. Introduction

Breast milk is the best nutrition for the newborn, and provides all the essential nutrients and bioactive compounds such as oligosaccharides (natural prebiotics), immune cells and bacteria (natural probiotics) and their metabolites (natural postbiotics) in order to promote his/her development [1,2]. However, in some cases, breastfeeding might not be possible, and then, an infant formula is the best substitute for breast milk. Thus, manufacturers infant formulas aim to mimic the composition of breast milk by adding bioactive agents to their formulas such as prebiotics, probiotics and more recently, postbiotics [3,4].

Prebiotics are defined as non-digestible compounds that are selectively metabolized by the microorganisms in the gut, thus modulating the composition and/or activity of the gut microbiota, conferring a health benefit on the host [5,6]. In this regard, the short-chain galactooligosaccharides (scGOS) and long-chain fructooligosaccharides (lcFOS) mixtures are the most studied prebiotics in infant formula. [7]. Clinical research has demonstrated that scGOS and lcFOS supplements change the stool consistency and fecal microbiota composition making them more similar to that of breastfed infants [7,8,9]. In addition, during early life, the incidence of atopic dermatitis, allergy, and infections such as respiratory tract and gastrointestinal infections in babies fed with this type of infant formula were lower than those receiving formulas lacking these components [10,11,12,13,14,15,16].

Moreover, breast milk is also a source of live bacteria [1,17], thus, probiotic bacteria are often added to infant formula. Probiotics are defined as live, natural microorganisms, that when administered in adequate amounts, confer health benefits to the host [18,19]. The most commonly commercialized probiotic bacteria are strains from the genera *Bifidobacterium* and *Lactobacillus* [20,21,22]. Probiotics have the potential to modulate baby’s immune maturation and even to prevent or treat different diseases such as allergies, type 2 diabetes, diarrhea, respiratory infections, infant colic, ulcerative colitis, obesity and irritable bowel syndrome throughout life, their effects being strain specific [10,21,23,24,25].

Finally, postbiotics are described as a preparation of inanimate microorganisms and/or their components that confers a health benefit on the host [26]. Postbiotics are a safe and novel strategy to obtain the beneficial effects of probiotic bacteria without their possible disadvantages such as the rare case reports of probiotic-related infections and the fact that probiotics could express virulence factors and transfer antibiotic resistance genes to pathogenic bacteria in the gut [27,28,29]. The various procedures (heat, high pressure, ionizing irradiation or sonication) used to inactivate bacteria affect the microorganisms involved in the fermentation process differently and can modify the postbiotic composition and the host’s response to the postbiotic [21,30]. Postbiotics have attractive properties such as a favorable absorption, distribution and excretion abilities, safety dose parameters and longer shelf life [31]. Moreover, these properties might indicate a higher capacity to generate biological responses in different organs and tissues in the host [31]. The effects of specific postbiotics differ between individuals and also can depend on the temporal changes in gut microbiota composition [21]. Although the mechanisms of action of postbiotics still remain unclear [27], postbiotics can change the composition of gut microbiota and their function and many of their outcomes rely on microbial metabolites, organic acids, carbohydrates, proteins, lipids, cell wall components and other fermented products generated in the matrix [21]. Short-chain fatty acids (SCFA) are the major end products of gut microbiota activity and are associated with healthy gut microbiota composition and function as well as enhancement of mucosal immunity and intestinal barrier function [2,21]. Some of these postbiotics, such as the SCFA, are current components present in breast milk [2].

In recent years, the use of postbiotics as a nutritional strategy has increased and their effects have been studied both at preclinical and clinical levels [21]. It has been observed that postbiotics are useful in inflammatory diseases such as irritable bowel syndrome, colitis, gout, arthritis, atopic dermatitis and asthma [21,22,32,33]. Moreover, postbiotics could prevent or treat infectious diseases such as gastroenteritis, respiratory tract or enteric infections caused by *Escherichia coli* or *Salmonella enteritidis serovar*, *Listeria monocytogenes* and *Escherichia coli K1* infection [27,34,35,36]. Finally, other uses of postbiotics could be in neurological and cardiometabolic disorders [37,38]. 

For a long time, literature mainly focused on the beneficial effects of prebiotics and probiotics in infant milk formula, but after the appearance of postbiotics in this field the advantages compared to probiotics and their many applications have been identified. Therefore, the present study aimed to examine the effects of a daily supplementation with a formulation with a postbiotic and prebiotic mixture, as happens in breast milk. The postbiotic is comprised of an inactivated fermented milk infant formula obtained from *Bifidobacterium breve* and *Streptococcus thermophilus* activity by an innovative fermentation process (Lactofidus™). Although these microorganisms are not found in breast milk, they are known to produce 3-galactosyllactose (3-GL), a human milk oligosaccharide (HMO) present in breast milk, and frequently used in pediatric population [2]. The prebiotic is based on a mixture of scGOS and lcFOS. The products alone or in combination were administered to healthy suckling rats. This study aims to elucidate the specific effects of this particular postbiotic, but also to ascertain whether its combination with prebiotics, which is also observed in breast milk, modifies the health outcomes in rat pups. For that, some growth and immune variables, microbiota composition and SCFA production, among others, were evaluated.

## 2. Materials and Methods

### 2.1. Animals

Pregnant Lewis rats (G14) provided by Janvier (Le Genest St Isle, France) were individually housed in cages (2184L Eurostandard Type II L, Tecniplast, West Chester, PA, USA), monitored daily and allowed to deliver at term. The cages contained bedding of large fibrous particles (Souralit 1035, Bobadeb S.L., Santo Domingo de la Calzada, Spain) and tissue papers (Gomà-Camps S.A.U., La Riba, Spain) as cage enrichment. The day of birth was registered as day 1 of life. On day 2, litters were randomly assigned to four experimental groups and were unified to 8 pups per lactating dam with a similar proportion (40–60%) of each sex in each litter. Pups had free access to maternal milk and rat diet. Dams were given a commercial diet corresponding to the American Institute of Nutrition 93 G formulation [39] (Teklad Global Diet 2014, Envigo, Indianapolis, IN, USA) and water ad libitum. Animal handling was performed during the first hours of the light phase on a scheduled basis, to limit the disturbance and biological rhythms’ influence. After separating all the mothers and keeping the pups in the home-cage, handling and oral administration was performed once a day. Afterwards, the dam was reunited with her litter. Animals were housed under controlled conditions of temperature (20–24 °C) and humidity (40–60%) in a 12 h light−12 h dark cycle (lights on at 8:00 a.m and lights off at 8:00 p.m), at the Faculty of Pharmacy and Food Science animal facility (University of Barcelona, Spain). Cage cleaning was performed weekly. All experimental procedures were conducted in accordance with the institutional guidelines for the care and use of laboratory animals and were approved by the Ethical Committee for Animal Experimentation of the University of Barcelona and the Catalonia Government (CEEA/Ref. 255/18 and PAMN/Ref.10176, respectively), in full compliance with national legislation following the EU-Directive 2010/63/EU for the protection of animals used for scientific purposes. Sample size estimation was calculated by the Appraising Project Office’s program from the Universidad Miguel Hernández de Elche (Alicante, Spain). The minimal number of animals to provide statistically significant differences among groups, using plasma immunoglobulin (Ig) G as a variable and assuming that there is no dropout rate and type I error of 0.05 (two-sided), was three litters per group, as in previous studies, because of the remarkable variability among litters [15,40,41,42].

### 2.2. Experimental Design and Sample Collection

Upon natural delivery, pups were distributed into four groups of 24 animals each (3 litters of 8 animals/group): the reference (REF) group and three groups supplemented with a mixture of scGOS and lcFOS (PRE), Lactofidus^TM^ (POST), and the combination of both (P+P). All supplementations were provided by Danone Nutricia Research (Utrecht, The Netherlands). 

Suckling rats were orally administered once daily, as previously described [10], with normalized volume/body weight of vehicle, prebiotic, postbiotic or their combination (9 µL/g/day), from the second to the sixteenth day of life, corresponding to the strict lactation period. The PRE group was supplemented with 0.8 g of scGOS/lcFOS per 100 g of body weight. GOS/FOS is a mixture of GOS (Vivinal GOS, Borculo Domo, Zwolle, The Netherlands) with a degree of polymerisation (dp) of 3–8, and long-chain FOS (Raftiline HP, Orafti, Wijchen, The Netherlands; average dp > 23) in a 9:1 ratio.

The POST group received 0.92 g/100 g body weight of an infant formula that contained heat inactivated milk fermented by the bacteria *Bifidobacterium breve* and *Streptococcus thermophilus* (Lactofidus^TM^) [43,44,45,46]. Particularly, the Lactofidus^TM^ process is a well-controlled fermentation process in which the two unique and proprietary strains mentioned before are used to ferment a milk matrix. During fermentation, both strains are metabolically active and produce bioactive compounds (postbiotics). After the fermentation process the milk matrix is spray dried and then used in the present study.

The P+P group were fed with both products at the same doses as when given separately and maintaining the volume of administration (9 µL/g/day). Finally, a matched volume of water was administered to the REF group. The product dose selections were based on previous studies with similar approaches [10,12,41,42,47].

Body weight was recorded daily. Moreover, the naso-anal and tail lengths were measured to determine the body/tail ratio. In addition, body mass index (BMI) was calculated as body *weight/lenght^2^* (g/cm^2^), and the Lee Index was calculated as (*weight^0.33^/length*) × 1000 (g^0.33^/cm). 

During the study, fecal samples were obtained after gentle abdominal massage, to determine changes in consistency and fecal weight. Stool consistency was scored from 1 to 4 in a blinded manner based on texture and amount as described: normal (1); soft (2); totally loose (3); and high amount of watery (4) feces. The sampling was performed always at the same time, after the pups were separated from their mothers and weighted, and prior to its administration.

Animals were euthanized at two different time points by randomly selecting 4 pups from each dam maintaining sex equality on each day: half of the litter on day 8 and the other half on day 16, to obtain tissue samples each time. Rats were intramuscularly anesthetized with ketamine (90 mg/kg) (Merial Laboratories S.A., Barcelona, Spain) and xylazine (10 mg/kg) (Bayer A.G., Leverkusen, Germany) and exsanguinated. Plasma was obtained in order to determine the Ig pattern. The weight of liver, stomach, spleen, thymus, small intestine and large intestine was also recorded. Moreover, the length of small and large intestines was measured. Then, a 1 cm central portion of the small intestine was immediately conserved in RNAlater® (Ambion, Applied Biosystems, Austin, TX, USA), incubated at 4 °C overnight and stored at −20 °C until PCR analysis. Moreover, cecal content was obtained on day 16 for the analysis of the microbiota composition and SCFA profile.

### 2.3. pH of Stools and Stomach Content

For pH determination, fecal samples from days 7–9 (middle of the study) and 14–16 (end of the study) were diluted in distilled water (up to 200 mg/mL) and gently agitated before the measurement. In contrast, stomach content samples from day 8 and 16 were measured directly without previous dilution. In both cases, pH was measured using a 5207 pH electrode for surfaces and a micropH 2001 pH meter (Crison Instruments, Barcelona, Spain).

### 2.4. Quantification of Immunoglobulins

At the end of the nutritional intervention, on day 16, plasma concentration of IgG1, IgG2a, IgG2b, IgG2c, IgM and IgA was quantified using ProcartaPlex™ Multiplex immunoassay (Thermo Fisher Scientific, Barcelona, Spain) as described in previous studies [12], in which specific color-coded capture beads were bound to the Ig of interest. Then, different detection antibodies conjugated to phycoerythrin (PE) were added. The specific concentration of each analyte was obtained by MAGPIX® analyzer (Luminex Corporation, Austin, TX, USA) at the Scientific and Technological Centers of the University of Barcelona (CCiT-UB). The sensitivity of the assay was as follows: 0.02 ng/mL for IgM; 0.78 ng/mL for IgG1; 0.02 ng/mL for IgG2a; 0.11 ng/mL for IgG2b; 0.19 pg/mL for IgG2c and 0.48 pg/mL for IgA. 

### 2.5. Gene Expression Analysis 

A 1 cm of a central portion of the small intestine of 16-day-old pups was homogenized for 30 s in lysing matrix tubes (MP Biomedicals, Illkirch, France) using a FastPrep-24 instrument (MP Biomedicals), as previously described [47]. After RNA isolation with the RNeasy® Mini Kit (Qiagen, Madrid, Spain) its purity and concentration was determined with a NanoPhotometer (BioNova Scientific S.L., Fremont, CA, USA). Later, the corresponding cDNA was obtained using thermal cycler PTC-100 Programmable Thermal Controller and TaqMan® Reverse Transcription Reagents (Applied Biosystems, AB, Weiterstadt, Germany).

The specific PCR TaqMan® primers (AB) used to assess gene expression with real-time PCR (ABI Prism 7900 HT, AB) were directed to the detection of IgA (4331348, made to order), barrier function molecules such as Muc2 (Rn01498206_m1, inventoried [I]), Muc3 (Rn01481134_m1, I) Ocln (Rn00580064_m1, I) and Cldn2 (Rn02063575_s1, I), Cldn4 (Rn01196224_s1, I) as well as to Toll-like Receptors (TLR), such as Tlr2 (Rn02133647_s1, I), Tlr3 (Rn01488472_g1, I) Tlr4 (Rn00569848_m1, I), Tlr5 (Rn04219239_s1, I), Tlr7 (Rn01771083_s1, I), Tlr9 (Rn01640054_m1, I), and maturation markers such as Fcgrt (Rn00583712_m1, I, encoding for FcRn), and Prdm1 (Rn03416161_m1, I, encoding for Blimp-1). The relative gene expression was normalized to the housekeeping gene Gusb (Rn00566655_m1, I) using the 2-ΔΔCt method [48]. Results were expressed as the percentage of expression in each experimental group normalized to the mean value obtained for the REF group, which was set at 100%, as in previous studies [40].

### 2.6. Microbiota Composition

DNA from samples of cecal content collected on day 16 (6 rats/group corresponding to 2 rats of each of the 3 litters constituting each experimental group) were amplified 25 PCR cycles. A negative control of the DNA extraction was included as well as a positive Mock Community control to ensure quality control. They were later sequenced in the V3-V4 variable region of the 16S rRNA gene. The Illumina Miseq sequencing 300 × 2 approach was assessed (Illumina Inc, San Diego, CA, USA) and sequences were merged and processed using MiSeq run and MiSeq Reporter (on-system software) in collaboration with (Microomics, Barcelona, Spain).

To estimate the alpha biodiversity, the number of observed operational taxonomic units (OTUs, i.e., richness), Pielou’s evenness and Shannon’s diversity indexes were calculated and to assess the beta diversity Unweighted Unifrac distance was measured. The taxonomic assignment of phylotypes was performed using a Bayesian Classifier trained with Silva database version 132- 99% OTUs full-length sequences [49]. The relative proportions of families and genera were calculated and represented with stacked bars. The category “others” represented in each graph includes those families whose presence was lower than 1% in the REF group and those genera whose presence was lower than 3% in the same group.

In order to study the presence or absence of taxonomic ranks (family and genera) in the experimental groups, Venn diagrams were created. A bacterial group was considered as present in the group when all 6 animals displayed proportions higher than 0.001%, while the bacterial groups detected in less animals were regarded as absent in the group.

### 2.7. Quantification of Short-Chain Fatty Acids in the Cecal Content

Cecal content samples of 16-day-old suckling rats were acidified in 0.1 M formic acid and homogenized using Pellet Pestles Cordless Motor (Sigma-Aldrich, Barcelona, Spain) to reach the concentration of 200 mg/mL to measure cecal SCFA levels by headspace-gas chromatography-mass spectrometry (HS-GC-MS) at the GC-MS unit of the CCiT-UB, as previously described [12]. Acetic, propionic, isobutyric, butyric, isovaleric and valeric acids were quantified. The lower limits of detection (μmol/g of feces) were as follows: 0.404 for acetic acid, 0.068 for propionic acid, 0.003 for isobutyric acid, 0.020 for butyric acid, 0.001 for isovaleric acid, and 0.001 for valeric acid.

### 2.8. Statistical Analysis

Results are expressed as mean ± SEM. The Statistical Package for Social Sciences (SPSS v22.0) (IBM, Chicago, IL, USA) was used for statistical analysis. Data were tested for homogeneity of variance and normality distribution by Levene’s and Shapiro–Wilk tests, respectively. When data were homogeneous and had a normal behavior, conventional one-way ANOVA test was carried out followed by the post hoc Bonferroni. Otherwise, the nonparametric Kruskal–Wallis test followed by the post hoc Mann–Whitney U test was performed. The Spearman correlation coefficient was used to search for correlation between gut microbiota and SCFA production.

With regard to microbiota composition, alpha diversity comparisons were carried out using Kruskal–Wallis test. Beta diversity distance matrices were used to calculate principal coordinates analysis (PCoA) and to make ordination plots using R software package version 3.6.0. Permanova and ANOSIM tests were used to determine the significance of groups present in community structure. Moreover, Permdisp test was used to identify location vs. dispersion effects [50]. Differential relative abundance of taxa was tested using two methods: ANCOM [51] and Kruskal–Wallis test. After Kruskal–Wallis test, Conover’s test with FDR Benjamini-Hochberg correction was added for pairwise comparison. Finally, Biodiversity R version 2.11-1, PMCMR version 4.3, RVAide Memoire version 0.9-7 and vegan version 2.5-5 packages were used for the different statistical analysis preformed. 

A principal components analysis (PCA) was performed with Simca v14.1 (Umetrics, Umea, Sweden) to analyze the natural clustering of samples. Two data matrices consisting of 40 rows and 8 variables (IgA, IgG, IgG1, IgG2a, IgG2b, IgG2c, IgM and Th1/Th2 ratio) were constructed in order to analyze the variance observed in the Ig profile, and 34 rows and 6 variables (TLR2, TLR3, TLR4, TLR5, TLR7 and TLR9) in order to analyze the variance observed in the TLRs’ expression. Data were represented in score plots. Significant differences were established when *p* < 0.05.

## 3. Results

### 3.1. Growth and Morphometry

**Figure 1:**
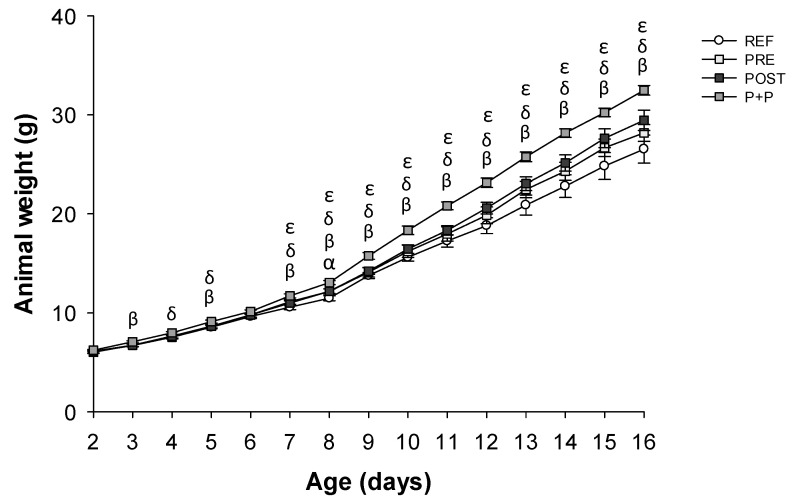
Body weight of pups (g) during the study (from day 2 to day 16 of life). Results are expressed as mean ± SEM (n = 24 animals/group). Statistical differences: α *p* < 0.05 PRE vs. REF, β P+P vs. REF, δ P+P vs. PRE and ε P+P vs. POST. REF: reference group; PRE: group supplemented with a mixture of scGOS and lcFOS; POST: group supplemented with Lactofidus^TM^; P+P: group supplemented with the combination of both.

As it can be observed in Figure 1, animals from the P+P group showed higher body weight than those from the REF, PRE and POST groups (*p* < 0.05). The highest differences were observed at the end of the study (day 16), when P+P showed an ~18% increase compared to the REF group. On day 8, a slightly increase in body weight was observed in the PRE animals compared to REF ones (*p* < 0.05). Finally, POST group had similar weights compared to those of REF (Figure 1). The BMI was not affected due to the diets (Table 1). 

Some differences were observed in the naso-anal, naso-tail, body/tail length ratio and Lee index on day 16 (Table 1. On day 16, prebiotic and postbiotic supplementation increased the naso-anal and the naso-tail measures compared to REF, PRE and POST animals (*p* < 0.05) (Table 1). Moreover, the PRE group showed a higher body/tail length ratio compared to REF, POST and P+P (*p* < 0.05) and a lower Lee index compared to REF group (*p* < 0.05). No differences were observed in the POST group compared to REF animals. 

In addition, on day 8, some of these changes were already present. Specifically, POST group showed a ~5% increase in naso-anal length and a ~2% reduction in Lee index compared to REF animals *p* < 0.05). P+P group had also a higher naso-anal length (~3.5%) and body/tail length ratio (~6%) compared to REF group (*p* < 0.05) and the Lee index was also higher (~2.5%) than that in the POST group (321.49 ± 1.72; *p* < 0.05). 

With regard to organ weights, as it can be observed in Table 1, on day 16, PRE and P+P groups showed a higher relative weight of the large and small intestine compared to REF and POST animals (*p* < 0.05). In addition, P+P group showed a higher relative weight of the liver and a lower large intestine length/weight ratio compared to REF group (*p* < 0.05). No differences were observed in the POST group compared to REF animals (Table 1).

On day 8, after a few days of supplementation, other changes also appeared. A ~20% increase in spleen relative weight was observed in PRE and P+P groups (compared to REF animals (*p* < 0.05). In addition, PRE group showed a 9–40% lower relative weight increase in liver compared to REF, POST and P+P animals *p* < 0.05), whereas in POST group it was higher compared to REF. All supplemented groups showed an increase in the relative small intestine weight, especially P+P group, which showed the highest value compared to all groups (*p* < 0.05), whereas POST showed the lower value of the supplemented groups (*p* < 0.05). In addition, P+P animals showed a 25% reduction in the length/weight ratio of the large intestine with respect to REF ones (*p* < 0.05). 

Only on day 16, the POST group showed higher platelets count compared to REF and PRE animals (*p* < 0.05), whereas PRE group showed a lower hematocrit compared to REF group (*p* < 0.05). P+P group had a reduction in the mean cell volume of erythrocytes compared to REF and POST animals (*p* < 0.05) (Supplementary Table S1). On day 8, the hematological variables in all supplemented groups were similar to REF ones (data not shown). 

### 3.2. Fecal and Stomach Content Variables

**Figure 2:**
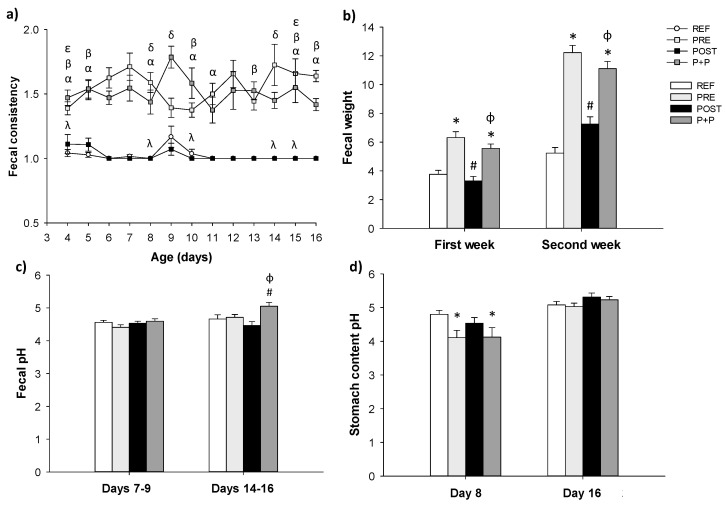
Fecal consistency during the study (from day 4 to day 16 of life) (**a**) and mean fecal weight from day 4 to day 7 (first week) and from day 8 to day 16 (second week) (**b**), fecal pH (on days 7–9 and 14–16) (**c**), and stomach content pH (on day 8 and 16) (**d**). Results are expressed as mean ± SEM (n = 12–24 animals/group). Statistical differences: α *p* < 0.05 PRE vs. REF, β P+P vs. REF, λ POST vs. PRE, δ P+P vs. PRE and ε P+P vs. POST (**a**), * vs. REF, # vs. PRE and ϕ vs. POST (**b**–**d**). REF: reference group; PRE: group supplemented with a mixture of scGOS and lcFOS; POST: group supplemented with Lactofidus^TM^; P+P: group supplemented with the combination of both.

Stool samples were collected and scored (scale 1–4) daily during all the studied period. The PRE and P+P supplementations induced changes in fecal consistency increasing the number of soft feces (*p* < 0.05). In contrast, the supplementation with the postbiotic did not affect the stool score (Figure 2a). 

As it can be observed in Figure 2b, during the first (from day 4 to day 7) and second (from day 8 to day 16) week of life, PRE and P+P groups had a higher fecal weight compared to REF and POST animals (*p* < 0.05). POST group showed similar fecal weight compared to REF ones, however, a tendency to increase the fecal weight was observed during the second week (*p* = 0.05).

The fecal pH and the stomach content pH were also measured (Figure 2c,d, respectively). P+P showed a higher fecal pH compared to PRE and POST groups at days 14–16 (*p* < 0.05). No differences in the fecal pH were observed between any of the supplemented groups and the REF animals, although a tendency to reduce the fecal pH in the PRE group was observed (*p* = 0.09) on days 7–9 and a tendency to increase the fecal pH in the P+P group was observed on days 14–16 (*p* = 0.08) (Figure 2c). Moreover, on day 8, PRE and P+P groups showed a reduction in the pH of the stomach content compared to REF animals (*p* < 0.05, Figure 2d). 

### 3.3. Immunoglobulins in Plasma

Plasma concentrations of IgG, IgM and IgA isotypes, IgG subclasses, as well as the Th1/Th2 ratio were quantified at the end of the study (day 16, Table 2). 

PRE group showed an increase in the percentage of IgM caused by a reduction in that for IgG (*p* < 0.05), although the percentage of IgG2b subclass was higher compared to REF animals (*p* < 0.05). POST group showed a higher percentage of IgM whereas the levels of IgA, IgG and the subclasses IgG1, IgG2a, IgG2b were lower compared to REF group (*p* < 0.05). Moreover, although the percentage of IgG and IgG2b were reduced (*p* < 0.05), the proportion of the IgG2c subclass was higher (*p* < 0.05) in animals from POST group than in REF animals. Finally, in line with the POST group, a higher percentage of IgM and a reduction in IgA, IgG, IgG1, IgG2a, IgG2b, IgG2c levels were observed in the P+P group compared to REF (*p* < 0.05). Overall, the PRE and POST groups showed a higher Th1/Th2 ratio. The Ig profile between supplemented groups was also different. POST and P+P groups displayed lower levels of IgA, IgG1, IgG2a and IgG2b compared to PRE (*p* < 0.05). The percentage of IgG2b was lower in POST group whereas IgG2c was higher compared to PRE animals. On the other hand, P+P showed lower levels of IgG and IgM whereas the percentage of IgM increased compared to PRE group (*p* < 0.05) and the levels of IgG2c decreased compared to PRE and POST groups and the percentage of IgG2b was higher compared to POST (*p* < 0.05). The overall impact of the diets on the Ig pattern, is clearly observed in a PCA representation: the POST and P+P groups were clustered differently to REF, whereas the PRE group was intermediately distributed (Supplementary Figure S1a).

### 3.4. Gene Expression

**Figure 3:**
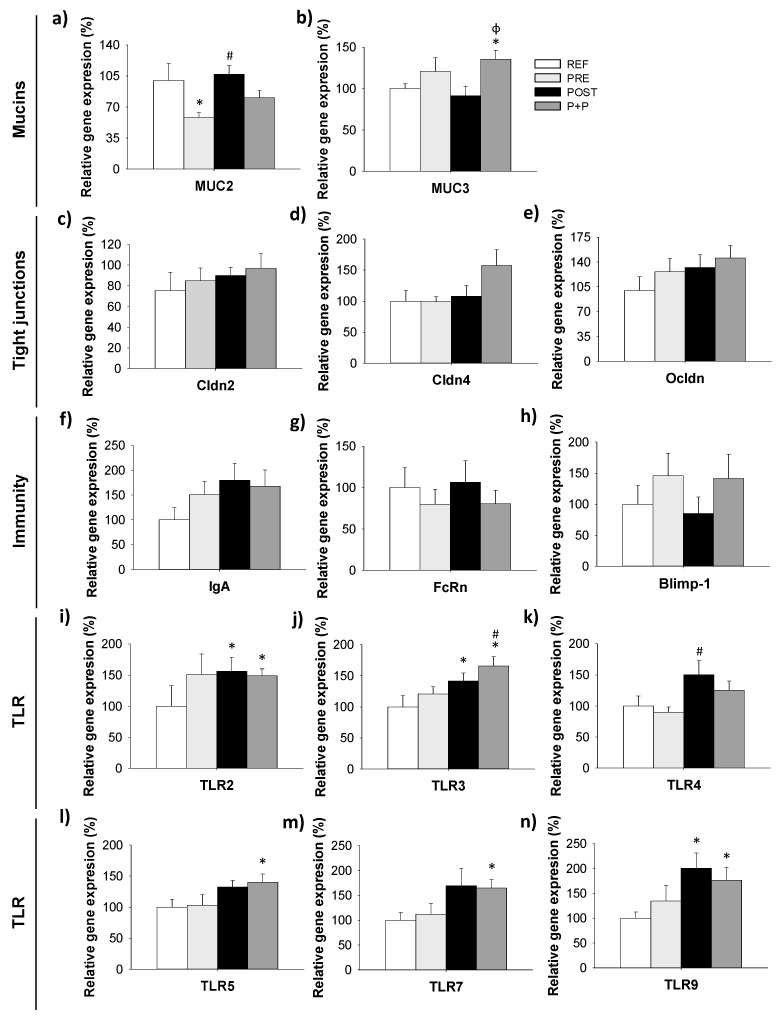
Relative gene expression of mucin (MUC) 2 (**a**) and MUC 3 (**b**), tight junction proteins claudin (Cldn) 2, Cldn4 (**c**,**d**, respectively) and occludin (Ocldn) (**e**), immunity related molecules IgA (**f**), FcRn (**g**) and Blimp-1 (**h**) and Toll-like receptors (TLR) 2 (**i**), TLR3 (**j**), TLR4 (**k**), TLR5 (**l**), TLR7 (**m**) and TLR9 (**n**) was quantified by real-time PCR on day 16. Relative gene expression was calculated with respect to REF animals, which corresponded to 100% of transcription. Results are expressed as mean ± S.E.M. (n = 8–9 animals/group). Statistical significance: * *p* < 0.05 vs. REF, # vs. PRE and ϕ vs. POST. REF: reference group; PRE: group supplemented with a mixture of scGOS and lcFOS; POST: group supplemented with Lactofidus^TM^; P+P: group supplemented with the combination of both.

The gene expression of molecules involved in the intestinal barrier capacity (mucin [MUC] 2, MUC3, claudin [Cldn] 2, Cldn4 and occludin [Ocldn]), intestinal immunity (IgA, FcRn and Blimp-1), and microbiota-host signaling receptors (TLR 2, 3, 4, 5, 7 and 9) were measured at the end of the supplementation period (on day 16) (Figure 3).

The gene expression of MUC2 was lower in the PRE group compared to REF and POST animals (*p* < 0.05. Figure 3a). As it can be observed in Figure 3b, the levels of MUC3 were higher in P+P group compared to REF and POST group (*p* < 0.05). Although no differences were observed in the expression of Cldn2, Cldn4 and Ocldn, in the P+P group there was a tendency to increase the levels of Cldn 4 (×1.5 times; *p* = 0.07) compared to REF animals (Figure 3c–e).

With regard to the expression of molecules involved in the immune system maturation and functionality, no differences were observed in the levels of IgA, FcRn and Blimp-1 (Figure 3f–h). However, POST group showed a tendency to increase the expression of IgA compared to REF group (×1.8 times; p = 0.07).

Finally, POST group showed higher levels of TLR2, TLR3 and TLR9 compared to REF animals being 1.5 times higher than REF animals (*p* < 0.05) and higher levels of TLR4 compared to PRE group (×1.7 times; *p* < 0.05). In addition, an increase in the expression of TLR2, TLR3, TLR5, TLR7 and TLR9 (×1.4–1.8 times) was observed in P+P group compared to REF group (Figure 3i–n) and higher levels of TLR3 compared to PRE group (×1.4 times; *p* < 0.05). No differences were observed in the PRE group, although, a tendency to increase the levels of TLR2 was observed (1.5 times; *p* = 0.07). Thus, it seems that most of the effects of the postbiotic are maintained in the mixture, as it can be observed in the PCA from Supplementary Figure S1b, in which the POST and P+P groups showed a cluster that differed from that of the REF; in contrast, PRE group formed an intermediate cluster.

### 3.5. Microbiota

**Figure 4:**
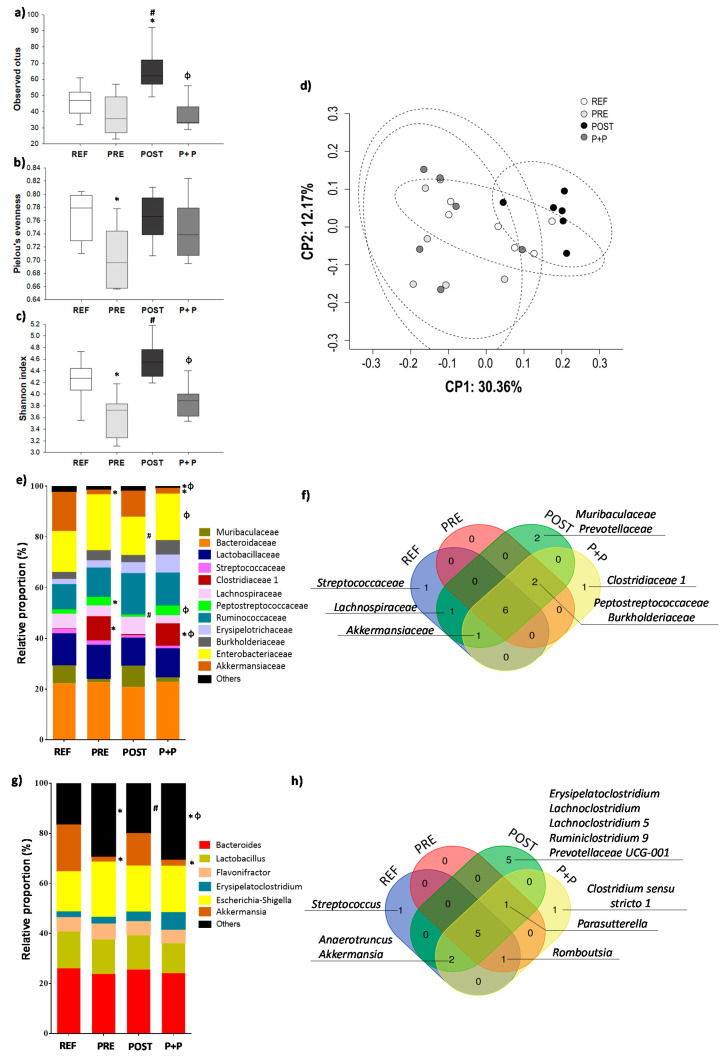
Assessment of cecal microbiota composition. The sequencing of the amplicon targeting the V3-V4 variable region of the 16S rRNA was performed using the Illumina Miseq sequencing 300x2 approach. The alpha diversity is represented by the richness (**a**), Pielou’s evenness (**b**) and Shannon’s indexes (**c**). The beta diversity was calculated measuring the Unweighted Unifrac distance (**d**). The taxonomic abundances of the main groups corresponding to family (**e**) and genera (**g**) are represented in stacked bars. The qualitative assessment of microbiota is represented in a Venn Diagram at the level of family (**f**) and genera (**h**). Results are derived from n = 6 animals/ group. Statistical significance: * *p* < 0.05 vs. REF, # vs. PRE and ϕ vs. POST. REF: reference group; PRE: group supplemented with a mixture of scGOS and lcFOS; POST: group supplemented with Lactofidus^TM^; P+P: group supplemented with the combination of both.

The cecal microbiota composition was analyzed on day 16 (Figure 4). The alpha diversity of microbial populations was assessed by richness, Pielou’s evenness and Shannon’s indexes. The group supplemented with the postbiotic had a higher richness compared to REF, PRE and P+P animals (*p* < 0.05; Figure 4a) and a higher Shannon’s index compared to P+P group (*p* < 0.05), whereas the PRE group showed a lower Pielou’s evenness (Figure 4b) compared to REF and a reduced Shannon’s (Figure 4c) index compared to REF and POST group (*p* < 0.05). The beta diversity calculated by measuring the Unweighted Unifrac distance (Figure 4d) showed that the POST group formed a cluster appart from the other groups.

The proportion of bacteria at the family level was different between groups. The rats supplemented with the prebiotic and with the combination of prebiotic and postbiotic showed a lower relative abundance of Akkermansiaceae and higher levels of Clostridiaceae 1 compared to REF animals (*p* < 0.05) and the levels of Clostridiaceae 1 in P+P group were also higher compared to POST group (*p* < 0.05). In addition, the relative abundance of Peptostreptococcaceae were increased in PRE group compared to REF and POST animals (*p* < 0.05) (Figure 4e). POST group showed lower levels of Enterobacteriaceae compared to PRE and P+P animals (*p* < 0.05). The minority families together constituting the group “others”, displayed some differences when analysed separately. Four rats from PRE group showed some levels of Bifidobacteriaceae (0.21 ± 0.11) compared to REF and POST group which did not have detectable values of this family in any animal (*p* < 0.05) confirming the bifidogenic effect of this prebiotic. In addition, the Chitinophagaceae proportion was lower in samples from POST group compared to those from the REF group (0.01 ± 0.01 and 0.11 ± 0.04, respectively; *p* < 0.05).

In order to gain a deeper understanding of the presence or the absence of bacterial groups after the different supplementations, Venn diagrams were created. The Venn diagrams were restrictive, and only when all the animals of the group had a count of that bacterial family, this family was considered to be present in the group. Therefore, Muribaculaceae and Prevotellaceae were only present in the POST group and Clostridiaceae 1 was present only in the P+P group. All the groups supplemented with the prebiotics, postbiotic or both of them, but not the REF group, showed the presence of the Peptostreptococcaceae and Burkholderiaceae families (Figure 4f).

The proportion of the majority genera was not statistically different between groups except for the reduction observed in the Akkermansia genus in the groups PRE and P+P compared to REF animals (*p* < 0.05) (Figure 4g). In the minority genera, no Bifidobacterium, Tyzzerella 3, Turicibacter and Enterobacter were found in REF nor POST, however some animals in the PRE group (four animals in Bifidobacterium, Tyzzerella 3 and Turicibacter and five animals, in Enterobacter) showed these genera (0.22 ± 0.12, 0.85 ± 0.40, 0.08 ± 0.03 and 0.11 ± 0.03, respectively; *p* < 0.05). Romboutsia abundance was higher in PRE and P+P group (3.31 ± 0.43, 3.81 ± 0.69, respectively) compared to REF group (1.58 ± 0.28; *p* < 0.05), whereas in POST group Romboutsia abundance was lower compared to the other groups (0.44 ± 0.05; *p* < 0.05). Vibrionimona proportions were reduced in POST compared to REF group (0.01 ± 0.01 and 0.12 ± 0.05, respectively; *p* < 0.05). In contrast to REF, PRE and P+P groups, Eubacterium nodatum group was found only in four animals from the POST group (0.27 ± 0.09; *p* < 0.05). The relative abundance of Clostridium ASF356 was higher in POST (0.70 ± 0.18) group compared to P+P animals (0.01 ± 0.01; *p* < 0.05). No animal from REF and PRE group showed levels of Clostridium ASF356. POST group had an increase in Faecalibacterium UBA1819 (1.35 ± 0.59) compared to PRE animals (0.03 ± 0.02; *p* < 0.05). No animal from REF and P+P groups showed levels of Faecalibacterium UBA1819. Anaerotruncus increased its proportion in P+P (8.36 ± 1.01) compared to REF group (2.91 ± 0.94, respectively; *p* < 0.05).

Regarding the Venn diagram information, POST group had the exclusive presence of the genera Erysipelatoclostridium, Lachnoclostridium, Lachnoclostridium 5, Lachnoclostridium 9 and Prevotellaceae UCG-001. Moreover, P+P group was the only one that showed Clostridium sensu stricto 1. Finally, Parasutterella was observed in the animals supplemented with all the products tested but not in the REF group (Figure 4h). 

### 3.6. Cecal SCFA Production

**Figure 5:**
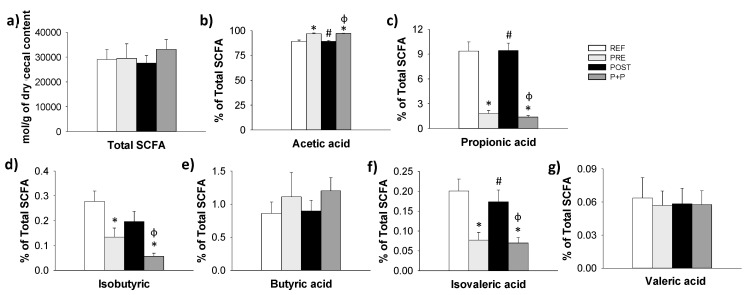
SCFA production in cecal samples of 16-day-old suckling rats. Total SCFA (**a**), acetic (**b**), propionic (**c**), butyric (**d**), isobutyric (**e**), isovaleric (**f**) and valeric (**g**) acid proportion (µmol/g of cecal content). Results are represented as mean ± SEM (n = 8–12 samples/group). Statistical significance: * *p* < 0.05 vs. REF, # vs. PRE and ϕ vs. POST. REF: reference group; PRE: group supplemented with a mixture of scGOS and lcFOS; POST: group supplemented with Lactofidus^TM^; P+P: group supplemented with the combination of both.

The amount of total SCFA and acetic, propionic, isobutyric, butyric, isovaleric and valeric acid proportion in the cecum of the suckling rats were measured at the end of the study (Figure 5). 

The total SCFA levels in the caecum was similar between groups (Figure 5a). However, the percentage of acetic acid was higher in PRE and P+P groups compared to REF and POST animals (*p* < 0.05) (Figure 5b). On the other hand, the proportion of propionic, isobutyric and isovaleric acids was lower in PRE and P+P groups compared to those in the REF and POST groups (*p* < 0.05). No differences were observed in the percentages of butyric and valeric acids between groups.

The acetic acid levels correlated positively with the relative proportion of Clostridiaceae 1 (r = 0.80, *p* < 0.05) (Clostridium sensu stricto 1 genus) Bifidobacteriaceae (r = 0.43, *p* < 0.05) (Bifidobacterium genus), Peptostreptococcaceae (r = 0.78, *p* < 0.05) and Enterobacteriaceae (r = 0.68, *p* < 0.05) families and negatively with Akkermansiaceae (r = −0.56 *p* < 0.05), Lachnospiraceae (r = −0.57, *p* < 0.05) Family XII (r = −0.49 *p* < 0.05), Muribaculaceae (r = −0.70 *p* < 0.05) and Prevotella (r = −0.69 *p* < 0.05) families. With regard to the propionic acid levels, a positive correlation was observed with the relative proportion of Akkermansiaceae (r = 0.56, *p* < 0.05), Prevotella (r = 0.69, *p* < 0.05), Family XII (r = 0.49, *p* < 0.05), Muribaculaceae (r = 0.62, *p* < 0.05) and Lachnospiraceae (r = 0.55, *p* < 0.05) families, whereas Clostridiaceae 1 (r = −0.80, *p* < 0.05), Enterobacteriaceae (r = −0.70, *p* < 0.05) Bifidobacteriaceae (r = −0.43, *p* < 0.05) and Peptostreptococcaceae (r = -0.72, *p* < 0.05) showed a negative correlation. Butyric acid correlated positively with Eggerthellaceae (r = 0.62, *p* < 0.05). Isobutyric acid correlated positively with the relative abundance of Akkermansiaceae (r = 0.58 *p* < 0.05) and isobutyric and isovaleric acids correlated negatively with Enterobacteriaceae (r = −0.67 and r = −0.55, respectively; *p* < 0.05) and Clostridiaceae 1 family abundance (r = −0.73 and r = −0.70, respectively; *p* < 0.05) whereas Muribaculaceae (r = 0.61 and r = 0.49, respectively; *p* < 0.05) Lachnospiraceae (r = 0.50 and r = 0.64, respectively; *p* < 0.05) and Prevotella (r = 0.53 and r = 0.49, respectively; *p* < 0.05) were correlated positevely (Supplementary Figure S2).

## 4. Discussion

There is evidence supporting the beneficial effects of prebiotics, postbiotics and probiotics, modulating the gut microbiota [11,20,35,37]. Formulas with postbiotics and prebiotics have been shown to support normal infant growth and are safe and well-tolerated in healthy infants [45,52,53]. In addition, prebiotics and probiotics have been demonstrated to result in stool consistency and frequency closer to breast-fed infants. Moreover, decreasing stool pH, increasing SCFA and modulating gastrointestinal microbiota towards a breast-fed infant profile [21,54,55]. In this study, we analysed the effects of a postbiotic (Lactofidus™) and its combination with a prebiotic mixture usually used in infant formulas (scGOS/lcFOS) in suckling rats, as a preclinical model for studying the immune system maturation.

In our study, the P+P group, but not the prebiotic or the postbiotic alone, showed a consistent higher growth rate during the suckling period compared to REF animals. In previous studies using this animal model, the administration of the same prebiotic mixture of scGOS/lcFOS alone—without the postbiotic—also showed this lack of effect on body weight [10] or just a slight increase at the end of suckling period [12]. Additionally, Morel F.B et al. did not observe an effect on growth and body weight of pre-weaning rats after GOS-Inulin supplementation [56]. With regard to humans, whereas a supplementation with GOS-FOS in early life showed an increase in the body weight and length of infants [13], other studies did not find these effects [9,45]. Focusing on the impact of the postbiotics used alone in growing infants, no effect has been previously observed [4]. In line with this, the studies evaluating infant weight/growth using a combination of scGOS/lcFOS with Lactofidus™ also demonstrated adequate growth [52] or even an impact on the infant’s length [57]. The possible rationale for this growth promoting effect in this preclinical study only when scGOS/lcFOS and the postbiotic are combined may involve a potentiation effect of the postbiotic on the well described capacity of oligosaccharides to increase intestinal calcium absorption [58,59,60]. 

It is widely known that scGOS/lcFOS mixture can prevent constipation that can occur in formula-fed infants by inducing a softer stool consistency and making it more similar to that of breastfed infants [9,54,57]. This feature of softer stools is also observed in our study after the PRE administration. In contrast, the postbiotic supplement alone did not affect the stool consistency. However, the combination of both compounds maintained the consistency observed in the PRE group. In this regard, besides the literature reporting this effect by the prebiotic mixture scGOS/lcFOS [9,21,55,61] it has been shown that the infant formula enrichment with Lactofidus™ combined with scGOS/lcFOS also induces softer stool consistency [46,52]. Same effect was confirmed by Huet F. et al., who also observed that infant’s fecal consistency after Lactofidus™ supplementation was higher compared to scGOS/lcFOS alone or scGOS/lcFOS combined with different percentages of Lactofidus™ (15% or 50%) [57].

Changes in stool consistency may be accompanied by an increase in fecal water and pH alterations. However, in our study the fecal pH was not significantly affected by any of the interventions compared to that in non-supplemented animals, although the PRE supplementation had a tendency to reduce the fecal pH during the first week. Previous studies in our group with this same prebiotic mixture in suckling rats, although under an infective process, showed some acidification effect [42]. In humans, some studies also observed that scGOS/lcFOS supplementation reduced the fecal pH [9,52,61]. Huet F. et al. reported that the infant formula supplemented with Lactofidus™ induced an increase in the fecal pH [57] whereas Béghin L. et al. observed that fecal pH was lower in infants that received infant formula containing scGOS/lcFOS with or without a postbiotic [52]. Moreover, during the first week in our study, the prebiotic and also its combination with the postbiotic induced a reduction in the stomach content pH. Very few data are available regarding the effect of these compounds in this compartment neither in animals nor humans. The result reported here could suggest a positive effect on the prevention of pathogen entry into the gastrointestinal tract as it occurs in the case of the lower fecal pH [61,62]. 

The Ig profile changed in all supplemented groups, higher IgM proportion and lower IgG levels were observed leading to an increase in the Th1/Th2 ratio. During the first weeks of life, the main circulating Ig are maternal IgG which are even in higher levels than the developing infant production of IgG and IgM and finally IgA [63]. Despite the lower levels of IgG, the increase in the proportion of IgM due to the prebiotic, postbiotic and its combination administration in early life could indicate a positive effect on the maturation of the immune system. In addition, after all supplementations an increase in an isotype associated with the Th1 response (rat IgG2b) and a decrease in an isotype associated with the Th2 response (rat IgG1) thus leading to a Th1 promoting effect was also observed, thereby suggesting again an enhancement of the immune system maturation. This effect on Th1 response is also observed at the end of suckling period in a model of rotavirus infection in rats supplemented with the scGOS/lcFOS mixture [12]. In humans, the Th1/Th2 IgG subclasses are different from rats whereas IgG1, IgG2 and IgG3 are associated with a Th1 response, IgG4 is related to a Th2 response. Van Hoffen E. et al. observed that infants at risk for allergy fed with infant hypoallergenic whey formula enriched in scGOS/lcFOS, or in maltodextrin for comparison, had a significant reduction in the Th1 associated IgG subclasses (i.e., IgG1, IgG2 and IgG3 for human) [64]. To our knowledge, this is the first report of a postbiotic intervention indicating changes in the Ig profile similar to that induced by a prebiotic intervention. 

Intestinal mucins have a role in the innate defense through the limitation and the neutralization of invasive pathogens [65]. The two most abundant intestinal mucins are the secretory MUC2 and the membrane bound MUC3 [66]. The prebiotic intervention induced a reduction in the levels of MUC2 gene expression, in agreement with a reduction also observed in a gastrointestinal infection model in suckling rats supplemented with this prebiotic mixture [12]. However, both rat and mice supplementation with GOS or FOS later in life, did not allow to observe this effect on MUC2 [67,68]. *Akkermansia* species are mucin degrading intestinal bacteria [69], thus a possible explanation for the reduction in MUC2 in this study after the scGOS/lcFOS intervention could be the reduction in *Akkermansiaceae* family observed in the PRE group. In this regard, in the PRE group could not need to continuously replace the mucus layer because there were less *Akkermansia* using mucus as energy source. Alternatively, the combination of the prebiotics with the postbiotic (P+P group) increased the MUC3 gene expression levels, suggesting a reinforcement in the innate barrier function of the suckling rats. In this regard, although it has been described that rats supplemented with GOS or FOS do not show differences in the expression of MUC3 [67], there is no available literature regarding the influence of a postbiotic alone or in combination.

Tight junction proteins have an important role in the intestinal barrier function regulating the permeability of molecules and acting as a physical barrier [70]. After the different supplementations, no differences were observed in their gene expression which differs compared to other studies evaluating GOS supplementation effect on the jejunum of piglets [71]. In addition, the expression of the genes FcRn, IgA and Blimp, which are related to the immune maturation and regulation, was not modified due to the interventions. However, the intestinal gene expression of the TLRs, which play a role in sensing pathogen-associated molecular patterns and contributing to eliminate pathogens and establishing the adaptative immunity [72], were highly modulated by the postbiotic intervention, and its combination with the prebiotic mixture. Upregulation of TLR2, TLR3 and TLR9 gene expression due to the postbiotic intervention was observed in the combination of the postbiotic and the prebiotic. In addition, only the combination was able to induce the increase in TLR5 and TLR7 expression, indicating that both types of compounds acting together are required to induce such an effect. To our knowledge, there is no literature about the expression of these genes after supplementation of a postbiotic alone or in combination with? in healthy rats or infants. However, this enhancement may suggest that the postbiotic, alone or in combination, can prepare the intestinal immune system for a better response against infections by strengthening the microorganism-host interaction.

In addition to the impact of these supplements on the immune system of the suckling rats, the microbiota composition and its functionality was evaluated. Although the number of samples per group (N = 6) could have been increased for clearer results, we found that the alpha diversity of microbial populations was able to be measured by three indexes, and whereas the intervention with the postbiotic induced a higher richness or number of observed OTUs, the prebiotic intervention led to lower Pielou’s and Shannon’s indexes, indicating lower overall biodiversity or lower equity in community proportion distribution. Although a FOS intervention in piglets affected diversity [73], very little is known about the postbiotics, in preclinical models or humans in this regard. Moreover, specific changes in microbial composition due to the interventions were observed in the PRE and the P+P group. The postbiotic alone did not change the microbial composition, however in combination it maintained the changes induced by the prebiotic supplementation in addition to some other slight differences. In this regard, the PRE and P+P groups showed a lower abundance of *Akkermansiaceae* family and *Akkermansia* genus compared to REF animals. *Akkermansia muciniphila* belongs to *Akkermansiaceae* family and this specie has been associated with health and inversely correlated to different diseases such as colitis and Crohn’s disease [74] or even Parkinson [75]. The reduction in the abundance of this bacterium could be due to the lower expression of MUC2 in both groups, specially the scGOS/lcFOS group. This reduction could decrease the formation of mucus and therefore the bacterium would have less substrate for growing. 

Moreover, some animal studies have demonstrated the promoting effect of FOS on *Akkermansia muciniphila* growth [76], thus the combination with GOS or the postbiotic and the early age could have a role in our differential result. In addition, some studies also showed an inverse correlation of *A. muciniphila* abundance with body weight [77,78], a fact that could be also involved in the growth promoting effect observed in P+P supplemented animals in our study. In addition, our results showed that all the interventions promoted the presence of *Peptostreptococcaceae* family in all the animals supplemented. Moreover, the abundance of this family was increased in the PRE goup with respect to the REF group. Very few data are available for this family and its modulation by diet, but it has been described to be in lower proportion in diabetic people [79] or in a depression induced rat model [80]. In addition, PRE and P+P groups showed higher abundance of *Clostridiaceae 1* family, in the P+P group mainly due to the presence of *Clostridium sensu stricto 1* genus as it was the only group in which all animals presented this genus. In contrast with our results, neonatal porcine supplemented with GOS showed a reduction in the abundance of *Clostridium sensu stricto 1* [81]. However, the higher abundance of these bacteria observed in our study due to the prebiotic supplementation may be involving some positive effect in the intestinal ecosystem because it is in line with lower abundance of this genus in diarrheal neonatal piglets [82]. The prebiotic intervention, but not the postbiotic or the combination confirmed the already described bifidogenic effect, which has also been described in previous studies in rat pups [56] and infants [9,57,61,83]. It should be taken into account that the relative abundances of bifidobacteria in this suckling rat model is much lower compared to the bifidobacterial levels observed in an infant, thus the early life microbiota response to these microbial-modulator products should be interpreted cautiously.

In our study, only the postbiotic group displayed *Prevotellaceae* and *Muribaculaceae* families and the genus *Prevotellaceae UCG-001*, *Erysipelatoclostridium*, *Lachnoclostridium*, *Lachnoclostridium 5* and *Ruminiclostridium 9* in all the animals supplemented. This particular growth promoting effect is of importance in basis of the current literature. On the one hand, the presence of *Prevotellaceae* in the microbiota of the postbiotic supplemented animals is in line with the observation that the supplementation with only FOS in piglets induced a higher relative abundance in *Prevotella* species, among others [73]. The healt promoting effect of this change is based on the fact that *Prevotellaceae UCG 001* is negatively correlated with markers of glucose and lipid metabolism disorders [84], and that patients with chronic kidney disease had lower *Prevotellaceae UCG-001* than healthy controls [85]. However, a spontaneous diabetes type 2 rat model showed higher levels of *Prevotellaceae UCG-001* compared with healthy Wistar rats [86]. On the other hand, although the higher abundance of *Muribaculaceae* in the POST group, which is described to be inversely associated with body weight [87,88], no effect was found in this regard in the present study. With respect to *Lachnoclostridium*, some species of this genus can protect mice against colitis [89], their abundance is lower in gastrointestinal tract neoplasia [90], even though higher levels of *Lachnoclostridium* were observed in adenoma [91]. Finally, *Ruminiclostridium 9,* also present after the POST intervention, are SCFA-producing microbes [92] associated with body weight regulation, obesity, inflammation and aging [92,93]. Overall, the biological relevance of these changes induced by the postbiotic in this context should be further elucidated. 

With regard to microbial functionality, SCFAs are the main products of microbial fermentation in the gut and play an important role in the interaction between diet, gut microbiota and host immune response [22,94]. SCFAs are considered anti-inflammatory mediators [22] with regulatory roles in energy homeostasis, glucose and lipid metabolism, and inulin sensitivity [31,37]. Although, none of the nutritional interventions modified the total amount of SCFAs, some qualitative changes were observed in the animals receiving the prebiotic mixture, alone or in combination with the postbiotic. Particularly, a profile of SCFAs with lower proportion of propionic, isobutyric and isovaleric acid and higher proportion of acetic acid was found. In accordance with our results, Frédéric Huet et al. observed that the presence of scGOS/lcFOS in an infant formula modulated the children microbiota and was associated with a change in SCFA pattern, which contained higher proportion of acetate and a lower proportion of propionate and other SCFAs [57]. Moreover, also in infants, the intestinal acetic acid production was increased in GOS supplemented formula making the profile more similar to that found in breastfed infants [83]. In agreement with our results, L. Beghin et al. also observed that isovaleric acid was reduced in infants fed with a fermented infant formula supplemented with scGOS/lcFOS, although they also observed changes in butyric acid [52] that we did not observe in our study. However, other similar approaches in early life, such as those using GOS in piglets showed an increase in butyric acid, among other changes [71,81].

To further study the changes observed in the relative abundances of gut microbiota and the modification in the pattern of SCFA production, the two variables were correlated. We observed that propionic acid production was correlated positively and negatively with some bacterial families in agreement with the literature [69,95]. The present study also showed some correlations regarding the acetic acid production, as described in other studies [96,97,98], as well with isovalerate and isobutyrate [99,100]. Overall, these correlations showed that the supplementation with scGOS/lcFOS and their combination with Lactofidus™ changed the relative abundances of gut microbiota and consequently modified the pattern of SCFA production.

## 5. Conclusions

The nutritional supplementation with the prebiotic (scGOS/lcFOS), the postbiotic (Lactofidus™) and the combination of both, modulated the Ig profile, but the prebiotic mixture and the postbiotic induced differential effects: whereas scGOS/lcFOS induced softer feces and modulated microbiota composition and SCFA profile, Lactofidus™ upregulated TLR gene expression. The use of the combination of scGOS/lcFOS and Lactofidus™ kept both effects observed separately, but also showed a synergistic impact on animal growth. Thus, the combined use of both products seems to be a good strategy to modulate immune and microbial features in early life.

## Figures and Tables

**Table 1:** Growth-associated measurements and relative weight of organs at the end of the study (day 16 of life).

	REF	PRE	POST	P+P
Naso-anal (Body, cm)	9.10 ± 0.16	9.44 ± 0.10	9.38 ± 0.10	9.75 ± 0.07 *# φ
Anus-tail (Tail, cm)	4.78 ± 0.11	4.58 ± 0.07	5.00 ± 0.07 #	4.99 ± 0.07 #
Naso-tail (cm)	13.88 ± 0.24	14.03 ± 0.16	14.38 ± 0.08	14.74 ± 0.13 *# φ
Body/Tail length ratio	1.91 ± 0.03	2.06 ± 0.02 *	1.88 ± 0.04 #	1.96 ± 0.02 #
Body mass index (g/cm^2^)	0.32 ± 0.01	0.32 ± 0.00	0.33 ± 0.01 #	0.34 ± 0.00 #
Lee index (g^0.33^/cm, ×1000)	327.35 ± 2.53	321.97 ± 1.00 *	329.16 ± 2.78	327.21 ± 1.42 #
Spleen/BW ratio (%)	0.43 ± 0.03	0.47 ± 0.02	0.47 ± 0.02	0.49 ± 0.02
Thymus/BW ratio (%)	0.45 ± 0.01	0.44 ± 0.01	0.45 ± 0.01	0.45 ± 0.01
Liver/BW ratio (%)	3.40 ± 0.05	3.47 ± 0.06	3.42 ± 0.10	3.54 ± 0.08 *
Large int./BW ratio (%)	0.60 ± 0.01	0.71 ± 0.02 *	0.57 ± 0.02 #	0.68 ± 0.02 * φ
Small int./BW ratio (%)	3.48 ± 0.09	5.32 ± 0.09 *	3.45 ± 0.07 #	5.06 ± 0.15 * φ
Large int. length/BW (cm/g)	24.91 ± 1.48	22.78 ± 0.77	21.63 ± 1.28	20.41 ± 0.54 *#
Small int. length/BW (cm/g)	134.96 ± 6.49	142.73 ± 6.53	127.35 ± 2.86 #	120.41 ± 5.72 #
Stomach/BW ratio (%)	0.76 ± 0.02	0.75 ± 0.02	0.70 ± 0.01 #	0.71 ± 0.01

Relative weight of organs was expressed as percentage (%) with respect to the body weight (BW) and growth-associated measurements are expressed as mean ± SEM (n = 8–12). Statistical significance: * *p* < 0.05 vs. REF, # vs. PRE and φ vs. POST. REF: reference group; PRE: group supplemented with a mixture of scGOS and lcFOS; POST: group supplemented with Lactofidus^TM^; P+P: group supplemented with the combination of both.

**Table 2:** Concentration of immunoglobulins in plasma at the end of the study (day 16).

Ig (µg/mL)	REF	PRE	POST	P+P
IgG	3117.81 ± 271.58	2968.14 ± 194.43	2341.86 ± 202.01 *	1985.85 ± 110.81 *#
	(98.64 ± 0.04%)	(98.52 ± 0.03% *)	(98.47 ± 0.06% *)	(98.41 ± 0.05% *)
IgG1	260.53 ± 25.42	208.69 ± 14.56	142.05 ± 15.73 *#	130.10 ± 8.56 *#
	(8.97 ± 1.06%)	(7.02 ± 0.11%)	(6.07 ± 0.42%)	(6.56 ± 0.23%)
IgG2a	551.53 ± 38.91	499.33 ± 28.96	394.47 ± 31.40 *#	363.66 ± 19.07 *#
	(18.01 ± 0.65%)	(17.35 ± 1.27%)	(17.01 ± 0.79%)	(18.49 ± 0.69%)
IgG2b	1193.30 ± 109.62	1255.98 ± 93.37	813.73 ± 78.19 *#	758.59 ± 46.86 *#
	(38.12 ± 0.45%)	(42.11 ± 0.65% *)	(34.61 ± 0.66% *#)	(38.15 ± 0.88% #φ)
IgG2c	1112.44 ± 131.36	1004.12 ± 84.37	991.60 ± 87.20	733.49 ± 52.67 *#φ
	(34.91 ± 1.21%)	(33.52 ± 0.94%)	(42.30 ± 0.35% *#)	(36.79 ± 1.39% φ)
IgM	23.02 ± 1.55	26.86 ± 2.18	23.21 ± 3.14	20.46 ± 1.23 #
	(0.74 ± 0.02%)	(0.89 ± 0.03% *)	(0.96 ± 0.07% *)	(1.02 ± 0.04% *#)
IgA	18.97 ± 1.05	17.68 ± 0.76	13.30 ± 0.81 *#	11.61 ± 0.96 *#
	(0.62 ± 0.02%)	(0.6 ± 0.02%)	(0.57 ± 0.02%)	(0.57 ± 0.02%)
Th1/Th2 ratio ^a^	2.81 ± 0.20	3.20 ± 0.22 *	3.34 ± 0.08 *	3.03 ± 0.14

The percentage of 100% of the isotypes corresponds to the sum of IgG + IgM + IgA and the percentage 100% of the subtypes of IgG corresponds to the sum of IgG1 + IgG2a + IgG2b + IgG2c. Results were expressed as mean ± SEM (n = 8–12). Statistical significance: * *p* < 0.05 vs. REF, # vs. PRE and φ vs. POST. ^a^ Th1/Th2 ratio refers to the relationship between IgG2b + IgG2c: IgG1+IgG2a. REF: reference group; PRE: group supplemented with a mixture of scGOS and lcFOS; POST: group supplemented with Lactofidus^TM^; P+P: group supplemented with the combination of both.

## Supplementary Materials

The following are available online at https://www.mdpi.com/article/10.3390/nu13092975/s1.
