# Supplementary material for: Effects of a Postbiotic and Prebiotic Mixture on Suckling Rats’ Microbiota and Immunity
Source: Nutrients. 2021 Aug 27;13(9):2975. doi: 10.3390/nu13092975 (PMC8469903; doi:10.3390/nu13092975)
Supplement: Supplementary file 1 [file nutrients-13-02975-s001.zip › nutrients-1318554-supplementary.pdf]

nutrients

MDPI

Supplementary Materials:

Supplementary Table S1. Hematological variables at the end of the study (day 16).

	REF	PRE	POST	P+P
WBC (10^9 cells/L)	2.17 ± 0.36	2.00 ± 0.22	2.33 ± 0.26	2.11 ± 0.24
LYM (%)	63.35 ± 5.70	65.97 ± 1.21	62.70 ± 2.34	65.96 ± 1.26
MID (%)	7.75 ± 0.82	7.19 ± 0.42	8.27 ± 1.27	7.45 ± 0.38
GRAN (%)	28.90 ± 2.80	26.84 ± 1.12	28.28 ± 1.62	26.59 ± 1.11
LYM (10^9 cells/L)	1.43 ± 0.22	1.27 ± 0.14	1.36 ± 0.11	1.30 ± 0.14
MID (10^9 cells/L)	0.14 ± 0.02	0.12 ± 0.02	0.18 ± 0.06	0.11 ± 0.02
GRAN (10^9 cells/L)	0.80 ± 0.14	0.62 ± 0.08	0.79 ± 0.11	0.93 ± 0.24
HGB (g/L)	103.67 ± 7.03	97.50 ± 6.52	95.63 ± 2.92	96.58 ± 5.59
HCT (%)	25.51 ± 0.49	23.28 ± 0.82 *	23.95 ± 0.63	24.69 ± 1.09
MCV (fL)	68.68 ± 2.70	64.79 ± 1.08	65.85 ± 0.58	64.01 ± 0.48*φ
MCH (pg)	28.08 ± 2.53	27.23 ± 1.95	26.35 ± 1.25	25.15 ± 1.47
PLT (10^9 cells/L)	477.58 ± 26.52	475.75 ± 28.92	573.38 ± 25.62 *#	510.50 ± 56.87
MPV (fL)	9.22 ± 0.32	8.35 ± 0.10	9.19 ± 0.39	8.73 ± 0.22

White Blood Cells (WBC), Lymphocyte (LYM), Monocytes, eosinophils, basophils, blasts and other precursor white cells (MID), Neutrophils, monocytes, eosinophils, and basophils (GRAN), Hemoglobin (HGB), Hematocrit (HCT), Mean Cell Volume (MCV), Mean Cell Hemoglobin (MCH), Platelets (PLT) and Mean Platelet Volume (MPV). Results were expressed ad mean ± SEM (n = 8-12). Statistical significance: * $p < 0.05$ *vs.* REF, # *vs.* PRE and φ *vs.* POST. REF: reference group; PRE: group supplemented with a mixture of scGOS and lcFOS; POST: group supplemented with Lactofidus™; P+P: group supplemented with the combination of both.

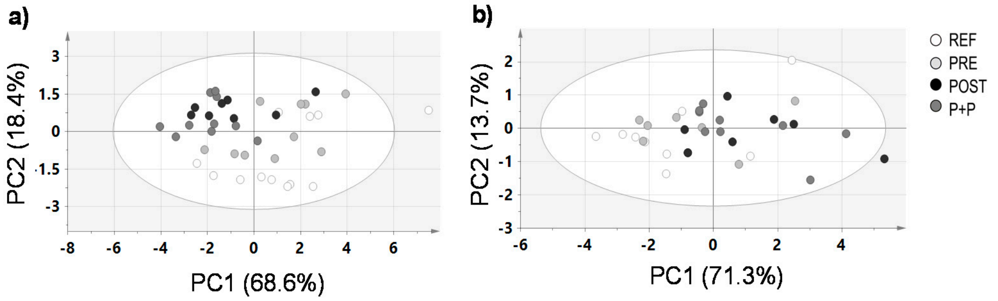

Supplementary Figure S1. Principal components analysis of the levels of immunoglobulins in plasma (**a**) and TLRs expression (**b**) on day 16 of pups' life. Results derived from n = 8. REF: reference group; PRE: group supplemented with a mixture of scGOS and lcFOS; POST: group supplemented with Lactofidus™; P+P: group supplemented with the combination of both.

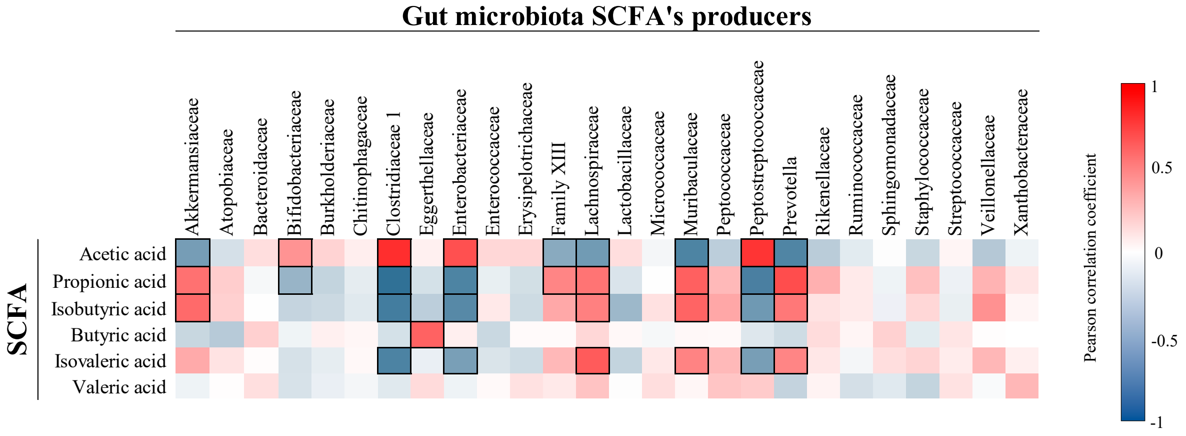

Supplementary Figure S2. Correlation between the SCFA and the SCFA gut bacteria producers. The Scheme 0. are shown in a bold frame. Results derived from *n* = 6. SCFA: Short chain fatty acids.
